# Screening of microalgae liquid extracts for their bio stimulant properties on plant growth, nutrient uptake and metabolite profile of *Solanum lycopersicum* L.

**DOI:** 10.1038/s41598-020-59840-4

**Published:** 2020-02-18

**Authors:** Chanda Mutale-joan, Benhima Redouane, Elmernissi Najib, Kasmi Yassine, Karim Lyamlouli, Sbabou Laila, Youssef Zeroual, El Arroussi Hicham

**Affiliations:** 1 Green Biotechnology laboratory, Moroccan Foundation for Advanced Science, Innovation & Research (MASCIR), Rabat Design Center Rue Mohamed Al Jazouli – Madinat Al Irfane Rabat 10 100, Rabat, Morocco; 2 AgBSprogram UM6P University Jorflasfar Morocco, Rabat, Morocco; 3 Microbiology and Molecular Biology Team, Center of Plant and Microbial Biotechnology, Biodiversity and Environment. Faculty of Sciences, Mohammed V University of Rabat, Avenue Ibn Battouta, BP 1014, Rabat, 10000 Morocco

**Keywords:** Metabolomics, Metabolomics

## Abstract

The present study investigates the biostimulant effects of 18 Crude Bio-Extracts (CBEs) obtained from Microalgae and Cyanobacteria on tomato plant growth, chlorophyll content, nutrient uptake and metabolite profile. Significant root and shoot length improvement (112.65%, 53.70%); was recorded at treatment with *Aphanothece* sp and *C. ellipsoidea* CBEs respectively. Meanwhile, the highest root and shoot dry weight (DW) (34.81%, 58.69%) were obtained at treatment with *Aphanothece* sp. The latter also displayed the maximum uptake of Nitrogen, phosphorus and potassium, which increased by 185.17%, 119.36% and 78.04% respectively compared with non-treated plants. Principal Component Analysis (PCA) confirmed that Phosphorus and Potassium levels in roots were closely related to enhanced Root length, whereas Nitrogen and chlorophyll b were closely related to Shoot and root DW. Additionally, Gas Chromatography-mass spectrometry (GC-MS) indicated that treatment with CBEs, induced the production of a vast array of metabolites. Treated plants recorded higher accumulation of palmitic and stearic acids, which could indicate a stimulation in *de novo* Lipid synthesis. CBEs also triggered the accumulation of pyridine-3-carboxamide (an amide active form of vitamin B3) and Linolenic acid; one of the key precursors in the biosynthetic pathway leading to plant jasmonates. Our results are a first step towards understanding the effects of microalgal extracts on plant physiology and biochemical pathways. Further investigations on biochemical fractionation of microalgal extracts and agronomic tests of their purified bioactive compounds could be a useful principal novelty for in-depth study of CBE action mechanisms. Other useful tools include; Comparative hormone profiling of treated and non-treated plants accompanied with combined High-Throughput Plant Phenotyping, transcriptomics and metabolomics analysis.

## Introduction

As the world population increases, concomitantly with the ecological impacts of climate change, agriculture is continuously faced with constraints, such as abiotic stresses, which severely hamper plant growth and development^1,2^. In order to maximize crop productivity and optimize plant growth conditions, conventional approaches such as irrigation, mechanization and the application of NPK fertilizers and biocides have been adopted^3^. However, due to the harmful impact of these intensifications on the environment and the ecosystem at large^3,4^, modern agriculture is challenged to adopt novel eco-friendly approaches. Today, the development of innovative technologies based on bioresources including plant biostimulants, have proven to be effective methods for improved crop performance. Plant biostimulants cannot only modify physiological processes to optimize productivity in crops^5^ but can enhance nutrient uptake, which ultimately optimize fertilizer consumption and use efficiency^6^. Thus, the use of plant biostimulants is a sustainable alternative strategy to improve crop yield and avert environmental pollution. A plant biostimulant is not a “fertilizer” in the true sense because it’s function and role in plant growth is independent of its nutrient content^7^, but it’s a material composed of substance(s) and/or microorganisms that stimulate natural processes in plants, leading to enhanced plant growth, nutrient use efficiency, tolerance against abiotic stresses and/or crop quality (European Biostimulant Industry Council EBIC, 2016)^5^. also defined plant biostimulants as; “*a formulated product of biological origin that improves plant productivity as a consequence of the novel, or emergent properties of the complex of constituents, and not as a sole consequence of the presence of known essential plant nutrients, plant growth regulators, or plant protective compounds”*. Among the various categories, seaweeds have so far proven useful for the production of plant growth biostimulants^7–9^. However, far less attention has been focused on Mmicroalgae and cyanobacteria in this field. Microalgae (eukaryotic) and cyanobacteria (prokaryotic), are unicellular microscopic photosynthetic organisms that grow in diverse aquatic habitats and even humid soils. Renewable and sustainable, microalgae and cyanobacteria are economical bioresources that have been exploited for several industrial applications as food ingredients, bioactive products and raw materials for biodiesel production^10^. Several microalgae and cyanobacteria species have exhibited remarkable pharmacological and biological qualities. The potential of these microorganisms as resources for bioactive compounds is attributed to their remarkable capacity to synthesise useful products such as polysaccharides, Proteins, Polyunasturated fatty acids, lipids and other bioactive metabolites, from atmospheric CO_2_^10^. Moreover, some studies have reported the presence of phytohormones (cytokinin, auxin, gibberellins, and brassinostreroids) in cellular extracts and growth medium of several microalgae species^11,12^. These phytohormones play key roles in plant growth and development. Also, microalgae products such as polysaccharides, can enhance plant growth and alleviate the effects of saline stress on plants^13–15^. In the present study, we investigated Crude Bio-Extracts (CBEs) of 18 Microalgae and Cyanobacteria species for their biostimulant effects on plant growth, nutrient uptake and metabolite profile of *Solanum lycopersicum* L. In addition, biochemical pathways of fresh and sea water organisms and subsequently their biochemical composition, may differ due to diverse adaptation mechanisms. Thus, a comparative study between the biostimulant properties of fresh and sea water Microalgae and Cyanobacteria was also highlighted. The aim of this research is to demonstrate the potential of microalgae as bioactive materials for bioproduct development in agriculture.

## Results

### Hydrolysis type and partial characterisation of the 18 shortlisted CBEs

CBEs of the 18 species of Microalgae and Cyanobacteria were extracted by acid hydrolysis at three different Sulfuric acid (H_2_SO_4_) concentrations (0M, 0.1M and 0.2M) and tested on plant growth parameters of tomato (*Solanum lycopersicum* L.) at three different doses (0.1, 0.5, and 1 g/L), under laboratory conditions. Therefore, a total of 162 Extracts were tested on plants, from where 18 CBEs were shortlisted based on their positive effects on plant development.

Partial characterization of the shortlisted CBEs revealed that they contained polysaccharides, soluble sugars, amino acids as well as diverse mineral elements such as nitrogen (N), phosphorus (P) and potassium (K). Table S1 shows the composition and hydrolysis type of the 18 shortlisted CBEs.

### Growth and Biomass concentration of the selected microalgae and Cyanobacteria species

All microalgae and cyanobacteria species showed growth increase during the 30-day culture period, no decline in cell density was recorded (Table 1). The mean growth rate, doubling times and biomass accumulation of all microalgae and cyanobacteria species were evaluated. Table 1 is presented in two groups for easier comparison: freshwater and seawater species of microalgae and cyanobacteria.

**Table 1:** Growth and Biomass concentration of the selected microalgae and cyanobacteria at 30-days old.

	Mean Growth rate ((µ) day^−1^)	Doubling time (days)	Biomass Concentration (gL^−1^)
**Cyanobacteria**			
*Aphanothese sp*.	0.283 ± 0.075	2.445	0.236
*Arthrospira maxima*	0.213 ± 0.103	3.253	0.886
*Arthrospira platensis*	0.201 ± 0.093	3.449	0.936
**Chlorophyta**			
*Chlorella pyrenoidosa*	0.159 ± 0.054	4.359	0.598
*Chlorella vulgaris*	0.224 ± 0.050	3.089	0.548
*Chlorella ellipsoidae*	0.154 ± 0.034	4.486	0.49
*Chlorella sorokiniana*	0.205 ± 0.043	3.376	0.562
*Chlorella marina*	0.128 ± 0.049	5.421	0.237
*Scenedesmus dimorphus*	0.225 ± 0.082	3.072	0.59
*Scenedesmus obliquus*	0.225 ± 0.029	4.688	0.979
*Chlamydomonas reinhardtii*	0.264 ± 0.086	2.625	0.574
*Dunaliella salina*	0.115 ± 0.035	5.983	0.736
*Tetraselmis marina*	0.137 ± 0.030	5.061	0.735
*Tetraselmis* sp.	0.108 ± 0.021	6.383	0.506
*Tetraselmis suecica*	0.111 ± 0.022	6.219	0.4
**Rhodophyta**			
*Porphyridium* sp.	0.245 ± 0.073	2.82	0.283
**Haptophyta**			
*Isochrysis galbana*	0.131 ± 0.046	5.255	0.643
**Ochrophyta**			
*Nannochloropsis gaditana*	0.127 ± 0.025	5.473	0.39

The growth rate is represented as the mean of 3 replicate cultures ± the standard error (n = 3), doubling times were evaluated from the mean growth rate.

Cyanobacteria and freshwater microalgae species exhibited the highest growth rates, ranging from 0.093 d^−1^ to 0.135 d^−1^. Whereas, seawater microalgae species exhibited lower growth rates varying from 0.067 d^−1^ to 0.096 d^−1^. Additionally, the final biomass concentrations of microalgae and cyanobacteria greatly varied from one specie to another.

### Effect of CBEs on tomato root growth under laboratory conditions

**Figure 1:**
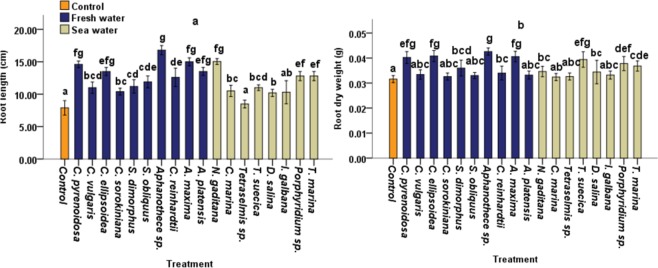
*Effects of CBEs* applications on (**a**) root length (**b**) root DW of 40 days old tomato plants. Data represent means and standard errors (error bars) of five biological replicates. Different letters indicate significant difference (p < 0.05), according to Turkey, One-way ANOVA.

To investigate the effect of Microalgae and Cyanobacteria crude extracts (CBEs) on tomato growth, the extracts were applied to tomato seedlings. Except for *Tetreselmis sp* and *I. gabana*, all CBEs displayed significant (p < 0.05) effect on root length compared with control plants (Fig. 1a). The highest percentage increases on root length (112.65%, 89.87%, 84.80%, and 70.88%) were recorded in plants treated with*, Aphanothece sp*., *A. maxima*, *C. pyrenoidosa* and *C. ellipsoidea* extracts of freshwater species respectively. Whereas the highest root length increase from seawater species was recorded at *N. gaditana* CBE treatment (90.38%) (Fig. 1a). Also, *D. salina, I. galbana* and *C. sorokiniana* CBEs exhibited the lowest effects on root length.

The maximum root dry weight (DW) increases (34.81%, 29.11%, 28.48% and 27.21%) were obtained respectively at *Aphanothece sp., C.ellipsoidea*, *A. maxima* and *C. pysrenoidosa* CBE treatments, for freshwater species while the highest root DW increase (24.68% and 19.62%) for seawater species was recorded respectively at *T. suecica* and *Porphyridium* sp. CBEs treatments (Fig. 1b).

### Effect of CBEs on shoot growth under laboratory conditions

**Figure 2:**
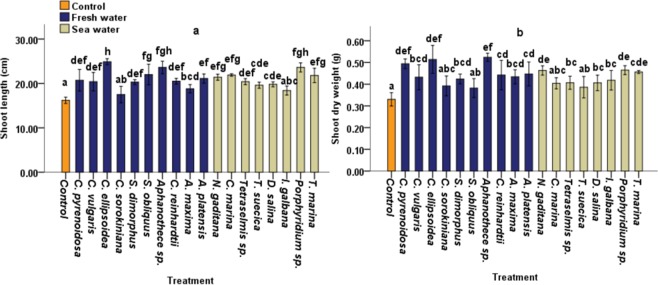
Effects of CBEs applications on (**a**) shoot length, (**b**) shoot DW of 40 days old tomato plants. Data represent means and standard errors (error bars) of five biological replicates. Different letters indicate significant difference (p < 0.05), according to a Turkey, One-way ANOVA.

CBE treatment exhibited relatively lower effects on shoot length as compared to root length. In the case of tomato plants treated with cyanobacteria and freshwater microalgae, we observed an increase of 53.6%, 45.68% and 35.8% in shoot length for *C. ellipsoidea*, *Aphanothece* sp. and *S. obliquus* CBEs respectively, compared to non-treated control plants **(**Fig. 2a). Whereas, highest increases in shoot lengths (45.67% and 35.18%) were recorded at treatment with *Porphyridium* sp. and *C. marina* CBEs respectively for seawater microalgae. No significant differences in shoot length were observed in tomato plants treated with *I. galbana* or *C. sorokiniana* extracts (Fig. 2a).

The highest increases in shoot DW (58.69%, 55.66% and 49.60%) for cyanobacteria and freshwater microalgae were obtained at treatment with *Aphanothece sp*, *C. ellipsoidea* and *C. pyrenoidosa* respectively, while seawater microalgae recorded a 40.70%, 40.22% and 38.09% increase at *Porphyridium sp., N. gaditana* and *T*. marina treatment respectively, compared with non-treated control plants (Fig. 2b).

### Leaf concentrations of photosynthetic pigments

**Figure 3:**
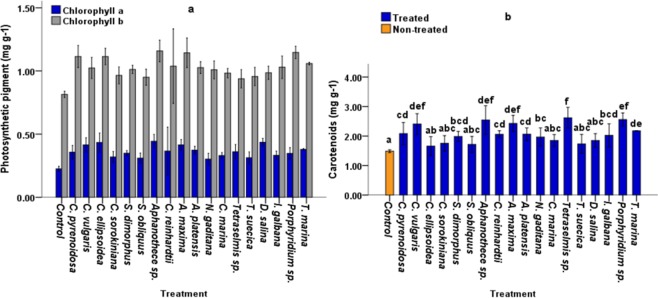
Leaf concentrations of photosynthetic pigments; chlorophyll a (mg g−1), chlorophyll b (mg g−1) (**a**); and Carotenoids (mg g−1) (**b**) in 40 days old tomato plants under laboratory conditions. five replicates were used. Different letters indicate significant difference (p < 0.05), according to a Turkey, One-way ANOVA.

Chlorophyll a, Chlorophyll b and Carotenoid concentrations in tomato leaves were evaluated 35 days after treatment. The results illustrated in Fig. 3a,b show a significant change in photosynthetic pigments in the majority of treated plants.

#### Chlorophyll a

CBEs displayed highest values at treatment with *Aphanothece* sp. and *C. ellipsoidea* extracts, which increased by 42.00% and 40.36% respectively compared with the control. Whereas the highest increase in Chlorophyll a content (40.63% and 36.70%) of seawater microalgae were obtained with *D. salina* and *Tetraselmis* sp crude extracts (Fig. 3a).

#### Chlorophyll b

A similar trend was observed in Chlorophyll b content, the maximum increase in chlorophyll b content (92.45%, 92.28% and 83.95%) across all treatments was observed in plants treated with *Aphanothece sp*., *A. maxima* and *C. pyrenoidosa* extracts respectively compared with non-treated control plants (Fig. 3a) while the highest Chlorophyll b content for seawater microalgae was observed at treatment with *Tetraselmis sp*. and *N. gaditana* extracts, which increased by 93.31% and 83.95% respectively compared to control plants (Fig. 3a).

#### Carotenoids

All CBEs displayed significant increases in carotenoid content (Fig. 3b) (p < 0.05). The highest values of the carotenoids content were obtained at treatment with *Tetraselmis sp, N. gaditana* and *Aphanothece* sp, extracts, which increased by 129.83, 124.75 and 123.05% respectively compared with control plants (Fig. **b**).

#### Conclusion

*On plant growth parameters, we did not observe a clear-cut difference between the bio-stimulatory effects of CBEs extracted from fresh and seawater Microalgae or Cyanobacteria. These results illustrate that the bio-stimulant properties of microalgae and cyanobacteria are independent of their water sources*.

### Root concentrations of Nitrogen, Phosphorus and Potassium

#### Nitrogen (N)

**Figure 4:**
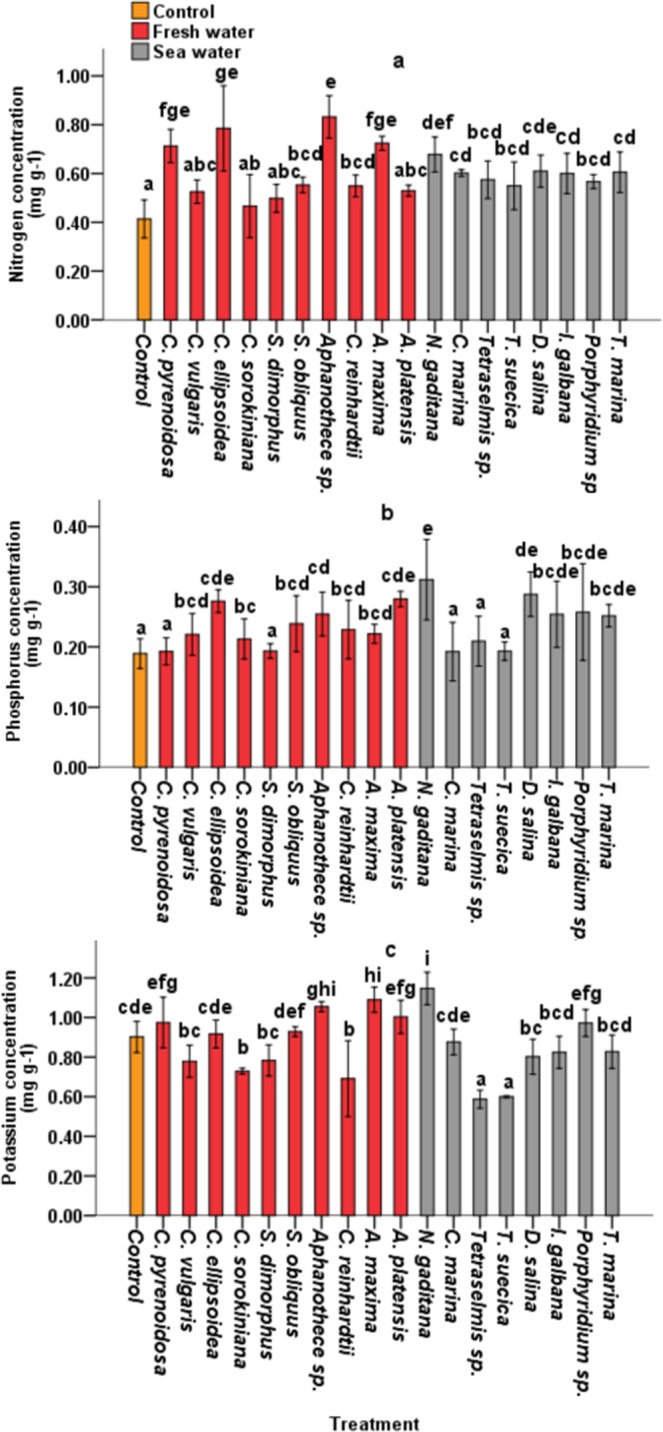
Root concentrations of Nitrogen (mg g^−1^) (**a**), Phosphorus (mg g^−1^) (**b**) and Potassium (mg g^−1^) (**c**) in 40-day old tomato plants under laboratory conditions. Five replicates were used. Different letters indicate significant difference (p < 0.05), according to a Turkey, One-way ANOVA.

Among the three macro elements, N was highly enhanced in all treated plants compared with non-treated plants. The highest N concentrations were recorded in plants treated with *Aphanothece* sp, *C. ellipsoidea* and *A. maxima* extracts, which increased by 101.07%, 89.79% and 75.04%, respectively compared to non-treated control plants. Whereas the lowest percentage increase (12.64%) was recorded at treatment with *C. sorokiniana* extracts (Fig. 4a).

#### Phosphorus (P)

Tomato plants treated with *Aphanothece sp, C. ellipsoidea* and *C. pyrenoidosa* extracts exhibited the highest P increase (65.08%, 52.15% and 48.11%) compared to control plants, whereas treatment with *S. dimorphus*, *C. marina* and *T. suecica* extracts, recorded the lowest effect on P concentration in tomato root biomass (1.72%, 2.13% and 4.54%) respectively (Fig. 4b).

#### Potassium (K)

CBE application had the least effect on K concentration in roots. (Fig. 4c) illustrated that the treatment of *C. sorokiniana*, *S. dimorphus, Tetraselmis sp*. and *T. suecica* extracts decreased K concentrations in roots by 16%, 20.53% and 27. 36.97% compared to non-treated control plants (0.902 mg g^−1^). The highest K levels were observed at treatment with *Aphanothece sp, C. ellipsiodea* and *T. marina* extracts which increased by 78.04%, 67.37% and 38.50% compared to the control.

### Treatment-variable Interactions through hierarchical clustering and PCA analysis

**Figure 5:**
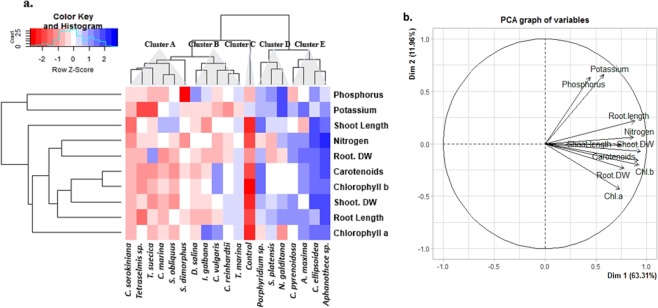
Hierarchical clustering and principal component analysis (PCA) to understand variable associations in treated tomato plants. CBEs were associated with five major clusters. (**a**) The entire data were analysed using PCA. The variables included root DW (root dry weight), shoot DW (shoot dry weight), Root length, Shoot length, Chl a (chlorophyll a), Chl b (chlorophyll b), Carotenoids (Carotenoids), Nitrogen, Phosphorus and Potassium. The lines originating from central point of PCA plot indicate positive or negative correlations of different variables (**b**); where their closeness indicates correlation strength with particular treatment S**3**.

Hierarchical clustering and principal component analysis (PCA) were performed to understand treatment-variable relationships in treated plants (Fig. 5b). The data of different parameters were normalized. The clustering analysis represented the normalized average mean values of all the studied parameters in both treated and control plants. PCA analysis revealed that Phosphorus and Potassium levels in root biomass were positively associated to Root length. This could indicate that Phosphorus and Potassium uptake was influenced by the Root length (Fig. 5b). PCA also showed that root Nitrogen concentration was more closely associated to Shoot DW, which was associated to chlorophyll b, indicating a correlation between Nitrogen uptake, chlorophyll b and shoot biomass accumulation in treated plants (Fig. 5b).

### Metabolomics using GC-MS approach

To further evaluate the effect of CBEs on plant growth and development, a comparative metabolite profile analysis of treated and non-treated control plants, was performed using GC-MS. CBEs effect on plant growth were clustered in 5 major groups (Fig. 5a). For GC-MS comparative metabolite analysis, 9 CBEs were randomly selected across the 5 cluster groups:

- Control plants
- *1 CBE (Tetraselmis* sp.) from cluster A (CBEs with the least effect on the majority of the studied parameters),
- *2 CBEs (D. salina* and *I. galbana)* from cluster B,
- *2 CBEs (Porphyridium* sp. and *N. gaditana)* from the cluster D.
- All CBEs from cluster E*; Aphanothece sp., C. ellipsoidea, A. maxima and C. pyrenoidosa* (CBEs with the highest on the majority of the studied parameters).

Treatment with CBEs, induced the production of a vast array of metabolites (Table. S6).

### Comparative metabolite profile of treated and non-treated control plants

**Figure 6:**
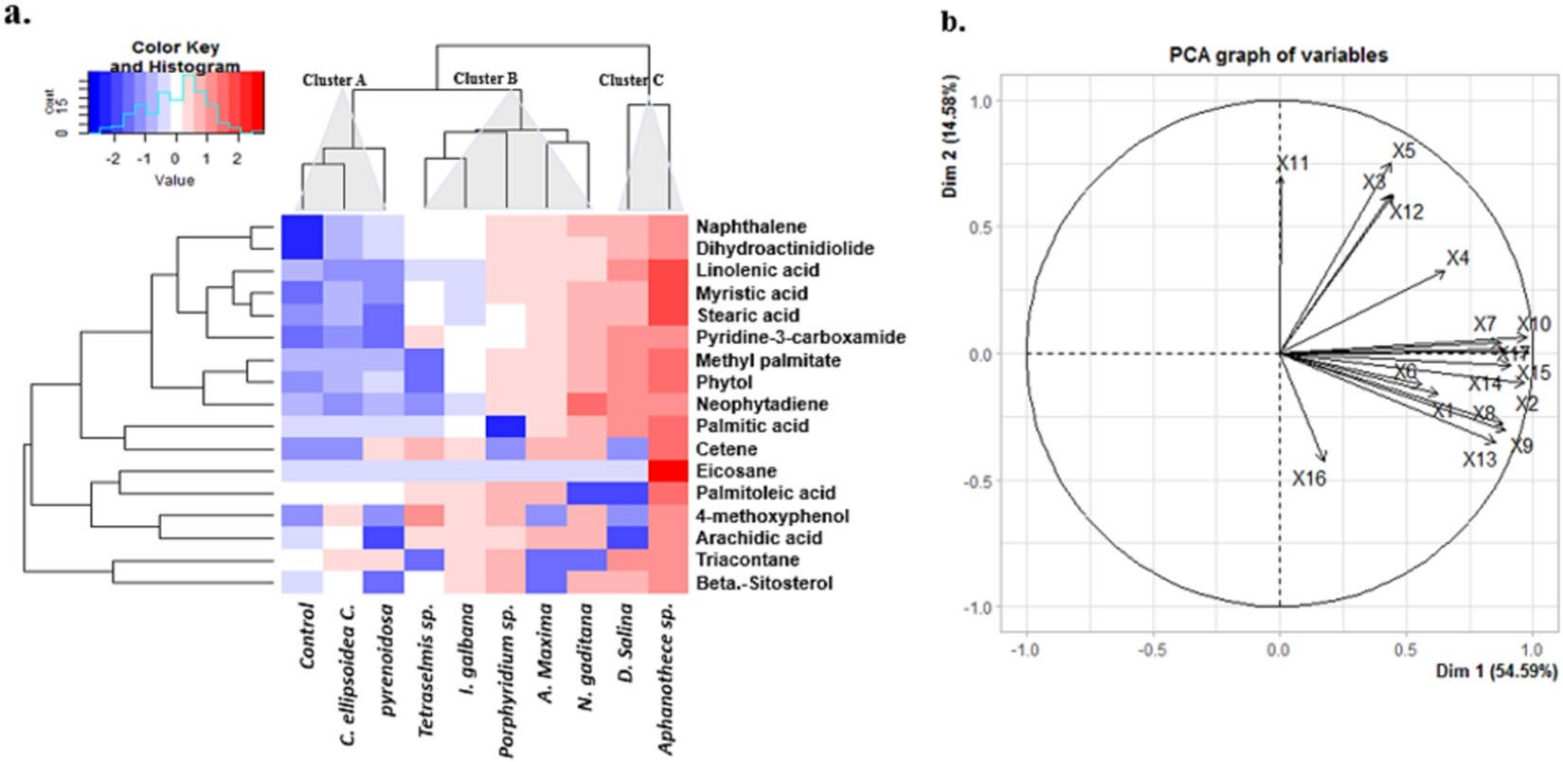
Hierarchical clustering and principal component analysis (PCA) of 17 metabolites detected in high concentration in 40 day old treated and non-treated tomato plants, under laboratory conditions. The Heat-map represents the normalized mean values (centered and scaled) of leaf metabolites of three replicates. The variables included: X1 = Beta.-Sitosterol, X2 = Linolenic acid, X3 = Cetene, X4 = Eicosane, X5 = Arachidic acid, X6 = Palmitic acid, X7 = Naphthalene, X8 = Neophytadiene, X9 = Methyl palmitate, X10 = Stearic acid, X11 = Palmitoleic acid, X12 = 4-methoxyphenol, X13 = Phytol, X14 = Pyridine-3-carboxamide, X15 = Myristic acid, X16 = Triacontane, X17 = Dihydroactinidiolide. Refer to Fig. S5 for variable -treatment b-Plot and Table S6 for all metabolites detected under different treatments.

Treatment with CBEs significantly influenced the profile of detected metabolites compared with the non-treated plants, a larger spectrum and higher concentration of metabolites was detected in all treated plants (Table. S6). Some metabolites including palmitic acid, stearic acid and linolenic acids, eicosane, phytol, pyridine-3-carboxamide were detected in significantly higher concentrations compared to non-treated control plants (Fig. 6). We observed the highest enhancement of palmitic acid (9372%, 4117%, 1539, 1110% and 1022%), stearic acid (12510%, 2635%, 2343%, 734% and 1137%) and linolenic acid accumulation (5185%, 2186%, 778%, 673% and 561%) at treatment with *Tetraselmis* sp, *D. salina*, *N. gaditana*, *Aphanothece* sp and *A. maxima* CBE respectively (Fig. 6). A similar trend was observed in phytol concentrations where the highest phytol enhancements (8947%, 5329%, 3310%, 2044% and 1973%) were recorded at Treatment with *Tetraselmis* sp, *D. salina*, *N. gaditana*, *Aphanothece* sp and *A. maxima* CBEs respectively (Fig. 6). The highest Pyridine-3-carboxamide enhancements (15227%, 10808%, 5440, and 3012%) were also recorded at treatment with *D. salina*, *Tetraselmis* sp, *N. gaditana*, and *I. galbana* CBEs respectively

#### Conclusion

*Although CBEs extracted from Tetraselmis sp. exhibited less effect on root and shoot sizes*, Fig. 6 *illustrates that Tetraselmis sp. CBE treatment recorded the highest effect on plant metabolite profile. Thus, the effect of plant biostimulants cannot only be evaluated on the basis of plant growth. Our results suggest that besides plant growth, some extracts trigger other biochemical processes such as the accumulation of lipophilic metabolites, which are related to plant abiotic stress tolerance and defence. The accumulation of Pyridine-3-carboxamide could also reflect more on crop quality as opposed to crop productivity. Therefore, microalgae and/or cyanobacteria based bioproducts could be orientated for diverse application purposes in agriculture*.

## Discussion

Our study demonstrated that CBEs can enhance growth and nutrient uptake in treated plants. Understanding the effects of CBEs on regulating physiological and biochemical pathways related to plant growth, nutrient uptake and Metabolomic profile can play a vital role in improving agriculture production. Our study is the first step towards such understanding.

The application of CBEs to tomato seedlings improved chlorophyll content, nutrient uptake from the soil and eventually, the root and shoot length and dry weight. Notably *Aphanothece* sp CBEs, significantly enhanced root length (112.65%), root and shoot DW (34.81% and 58.69%) respectively (Figs. 1 and 2b) while *C.ellipsoidea* CBEs played a significant positive role in improving the shoot length (53.70%) (Fig. 2a). Previous studies have shown that algal extracts and their purified compounds can induce strong physiological responses in plants and increase shoot and/or root weight^5,16–20^. In addition, microalgae extracts such as polysaccharides have proven to enhance growth of agricultural crops^13,15,21,22^. Sugars for instance act both as effective signalling molecules and as immediate substrates for intermediary metabolism, and could therefore promote plant growth and development^20,23^. A role for macro- and microelements, vitamins and phytohormones in alga extracts may also be suggested^11,12,20,24^. For instance^16^, reported that Potassium in seaweed extracts have a positive effect on regulating water status in treated plants and enhancing photosynthesis and meristematic growth. In the present study. CBEs contained various concentrations of sugars, proteins and NPK (Table 1). However, taking into consideration the CBE concentrations applied to plants, we could rule out the contribution of macroelements: Thus, the beneficial effects may involve several plant growth-promoters working synergistically.

N, P and K are three important macro elements frequently added as fertilizers in modern agricultural schemes. Therefore, this study targeted NPK concentrations in roots to evaluate the effect of CBEs on nutrient uptake. PCA analysis confirmed that improved P and K levels in roots was closely associated with enhanced root length (Fig. 5b), indicating that improved nutrient uptake observed in treated plants could also be attributed to increased root size. This is explained by the fact that enhanced root growth increases the root surface area and directly improves nutrient accessibility and uptake.

Treatment of plants with CBEs could also increase chlorophyll concentration in leaves (Fig. 3). The maximum increase in chlorophyll b content (92.45%, 92.28% and 83.95%) across all treatments were observed at treatment with *Aphanothece sp*., *A. maxima* and *C. pyrenoidosa* extracts for freshwater species respectively, and *Tetraselmis sp*. and *N. gaditana* extracts for seawater species, which increased by 93.31% and 83.95% respectively compared with control plants (Fig. 3). Our results are coherent with previous studies; which reported increased chlorophylls in plants treated with seaweed extracts. This increase in chlorophylls was attributed to reduced chlorophyll degradation and delayed plant senescence^7,25^. Increased chlorophyll content was also reported in *Brassica chinensis* L. treated with Kelp water extracts^20^. In addition, CBEs extracts might have several effects on photosynthetic pathways^26^. reported that genes involved in carbon fixation, including rubisco and carbonic anhydrase were upregulated by algal extracts. Besides chlorophyll content, improved nutrient uptake highly influence photosynthetic efficiency and consequently, plant biomass accumulation^27,28^. reported that N levels in plants is strongly correlated with photosynthetic rate while improved photosynthesis is recognized as one of the most efficient ways to increase nitrogen use efficiency and crop yield^29^. Additionally^30^, proposed that further increases in crop yield potential will rely in large part on improved photosynthesis. In this study, N concentration in root biomass was closely associated with Root DW, Shoot DW and Chlorophyll b (Fig. 5b), indicating that N taken up by roots was partly invested in photosynthetic machinery. Our results are coherent with^31^, who reported that the majority of assimilated N in plants is invested in photosynthetic machinery and is therefore fairly strongly positive correlated with photosynthetic rate^27,28^. Apart from directly taking part in photosynthesis, N also acts as an important regulator in manipulation of CO_2_ diffusion^32–34^.

Primary and secondary metabolite profiles are another indispensable component of crop performance, photosynthesis and biomass accumulation^35^. The major phytochemical groups detected by GC–MS analysis in this study included fatty acids, alkanes, alkenes, alcohols, terpenes, tocopherols and sterols (Fig. 6). Groups found in very high concentrations included fatty acids, alkanes, alkenes and alcohol terpenes. In the present study, phytols were the most abundant alcohol terpenes detected in all plants, with the highest phytol enhancement (8947%, 5329%, 3310%, 2044% and 1973%) recorded at Treatment with *Tetraselmis* sp, *D. salina*, *N. gaditana*, *Aphanothece* sp and *A. maxima* CBEs respectively (Fig. 6). Phytol is an acyclic diterpene alcohol and a constituent of chlorophyll that is generated from chlorophyll breakdown^36–38^. Phytols are essential in the biosynthesis of tocopherols which is a well-known lipid antioxidant and contribute to the protection of photosystem II (PSII) against photodamage under environmental stress^37,39^. On the other hand, a large proportion of phytol and *fatty acids* is converted into *fatty acid phytyl* esters in chloroplasts, which are involved in maintaining the integrity of the photosynthetic membrane during abiotic stress and senescence in *Arabidopsis thaliana*^40^. However, very high accumulation of phytyl fatty acids was only recorded in plants treated with *Aphanothece* sp and *A. maxima* CBEs (1088% and 1008%) respectively (Table S6). It could be suggested that the accumulated phytol observed in treated plants and coherent with chlorophyll content, could have been generated from chlorophyll breakdown during plant metabolite extraction. Phytol accumulation was also closely associated with palmitic acid (C16:0), linolenic acid (C18:3) and stearic acid (C18:0) concentration (Fig. 6). Increased palmitic C16:0 and stearic C18:0 acid accumulation in treated plants might indicate enhanced lipid biosynthesis. Literature has reported that the first stage of *de novo* lipid synthesis in plants commences with the generation of C16 and C18 fatty acids. These fatty acids are then elongated to very long chain fatty acids (VLCFAs), and mainly incorporated in sphingolipids (structural elements in lipid bilayers of the cell membrane) and cuticular wax layers^41,42^, thereby helping plants reduce water loss and pathogen invasion. In addition, our study demonstrated that all treated plants exhibited higher total fatty acid concentrations, indicating the biostimulant effects of CBEs on plants’ lipid profile.

CBEs also enhanced the accumulation of pyridine-3-carboxamide in all treated plants. Pyridine-3-carboxamide, also known as Nicotinamide or niacinamide is an amide active form of Vitamin B3^43^. Pyridine-3-carboxamide is the primary precursor of an essential coenzyme in ATP production known as nicotinamide adenine dinucleotide (NAD+), and the sole substrate of poly-ADP-ribose polymerase-1 (PARP-1)^43^. Today, Pyridine-3-carboxamide is commercially exploited in vitamin supplements and cosmetic products. In the present study, higher accumulation of metabolites such as pyridine-3-carboxamide in treated tomato plants indicates that CBEs may improve the biochemical composition and subsequently, the nutritional quality of crop products.

In conclusion, our study reveals the remarkable biostimulant properties of crude microalgae and cyanobacteria extracts. The comparative study between the biostimulant properties of fresh and seawater microalgae and cyanobacteria did not highlight any clear-cut differences, suggesting that the bio-stimulant properties of microalgae and cyanobacteria is independent of their water sources. PCA analysis illustrated a close relation between enhanced shoot DW and chlorophyll b content, indicating that shoot DW was attributed to improved photosynthetic capacity. In addition, CBEs also improved N uptake, which may contribute to an improved nitrogen metabolism and consequently enhance photosynthetic efficiency. In addition, our research demonstrated that treatment with CBEs induces the production of a vast array of plant metabolites. CBEs triggered the accumulation of linolenic acid (C18:3), one of the key precursors in the biosynthetic pathway leading to plant jasmonates, and pyridine-3-carboxamide which is an amide active form of vitamin B3. Treated plants also exhibited higher concentrations of palmitic (C16:0) and stearic (C18:0) acids, suggesting a stimulation in *de novo* Lipid synthesis. Some CBEs such as *Tetraselmis* sp., with the lowest effect on plant growth, triggered the production and accumulation of several plant metabolites, indicating that the effect of plant biostimulants cannot only be evaluated on the basis of plant growth, some extracts trigger other biochemical processes such as the accumulation of lipophilic metabolites, which are related to plant abiotic stress tolerance and defence.

The present study highlights the potential of CBEs for enhancing plant growth, nutrient uptake and metabolomic pathways. However not all microalgae species have significant biostimulant activities on plant growth parameters, suggesting that the biostimulant properties of CBEs are due to “specie-specific” metabolites produced by particular microalgae or cyanobacteria species. Our results are a first step towards understanding the effects of microalgal extracts on plant physiology and biochemical pathways. Further investigations on biochemical fractionation of microalgal extracts and agronomic tests of their purified bioactive compounds could be a useful principal novelty for in-depth study of CBE action mechanisms. Other useful tools include; Comparative hormone profiling of treated and non-treated plants accompanied with combined High-Throughput Plant Phenotyping, transcriptomics and metabolomics analysis.

## Materials and Methods

### Culture of microalgae

In this study, we used 18 Microalgae and Cyanobacteria species from the AlgoBioTech collection of the MAScIR Foundation (Moroccan Foundation for Advanced Science, Innovation and Research). we selected *Chlorella pyrenoidosa, Chlorella ellipsoidea, Chlorella vulgaris, Chlorella sorokiniana, Chlamydomonas reinhardtii, Aphanothece sp., Scenedesmus obliquus, Scenedesmus dimorphus, Arthrospira platensis, spirulina maxima, Dunaliela salina, Porphyridium sp., Tetraselmis suecica, Tetraselmis marina, Tetraselmis sp., Nanochloropsis gaditana, Isochrysis galbana* and *Chlorella marina*. The microalgae and Cyanobacteria species were cultivated in 3 replicates using 250 mL Erlenmeyer flasks containing culture mediums, according to the optimised standard culture medium protocols of MAScIR. The culture mediums were first selected based on the water source of Microalgae and Cyanobacteria. All softwater microalgae species and *Aphanothece* sp. were cultured in BG11 medium at pH = 7.2 whereas *Arthospira plantesis* and *Arthospira maxima* were cultured in Zarrouk’s medium at pH = 9.5. All sea water microalgae species were cultured in F2 medium at pH = 7.8, with the exception of Dunaliela salina, which was cultured in Walne’s medium. The culture mediums for all seawater species were made with sea-salt solution. The Microalgae and Cyanobacteria species were grown in their respective culture mediums at a standard temperature of 25 °C, 140 rpm of agitation and 135 µmol photons m^−2^ s^−1^ of continuous white fluorescent light. The harvesting of the Microalgae biomass was done by centrifugation at 6000 rpm or 5 minutes and the biomasses collected was dried at 50 °C for 7 days.

### Determination of microalgae growth characteristics

The growth of microalgae and cyanobacteria was monitored every 2 days by measuring the optical density of the culture at 680 nm, using a UV/VIS spectrophotometer. The initial optic density for all cultures was = 0.1 (±0.02). However, according to cell size and specific growth rates of Microalgae and Cyanobacteria species in the respective culture mediums used in this study, the final optic density and fresh biomass varied from one specie to another. The total fresh biomass for each specie, was harvested by centrifugation at 6000 rpm for 5 minutes and dried at 50 °C for 7 days.

The measured optical density was used to study the growth kinetics via the increase in density (in logarithmic scale) versus time. Growth rates were calculated using the following equations^44^:

1 $${\rm{G}}=\,\mathrm{ln}({\rm{C}}2/{\rm{C}}1)/({\rm{t}}2-{\rm{t}}1)$$

Where C1 and C2 indicates cell Concentrations at time 1 (t1) and time 2 (t2), respectively. Doubling time was also calculated once the specific growth rate was known.

2 $${\rm{Doubling}}\,{\rm{time}}=\,\mathrm{ln}\,2/{\rm{G}}$$

### Preparation of microalgae extracts by Acid hydrolysis

0.3 g dry biomass of each one of the 18 Microalgae and Cyanobacteria species was grinded in liquid nitrogen and hydrolysed in 20 mL of H_2_SO_4_ at three different concentrations (0M, 0.1M and 0.2M). The mixtures were then heated for 2 h at 95 °C with constant stirring (interrupted every 30 min by; 1 min vortexing and 10 min sonification). The resulting slurries were then autoclaved at 121 °C for one complete cycle. After the acid hydrolysis, the samples were cooled to room temperature, centrifuged for 10 min at 4 °C and 4000 rpm. The supernatants were collected as microalgae crude extracts and stored at −20 °C.

The crude bio-extracts (CBEs) of microalgae and Cyanobacteria were each tested on plant growth parameters of tomato (*Solanum lycopersicum* L.) under laboratory conditions at three different concentrations of CBEs (0.1, 0.5, and 1 g/L).

Therefore, a total of 162 crude extracts were tested on plants: The effect of the 162 CBEs was tested on tomato seedlings. For each microalgae and cyanobacteria specie, 9 different extracts based on the H_2_SO_4_ hydrolysis concentration (0M, 0.1M and 0.2M) and application dose (0.1, 0.5, and 1 g/L) were tested. For each specie, only 1 out of 9 tested extracts was retained, based on their positive effects on plant development. Therefore, a total 18 CBEs were shortlisted

The 18 selected CBEs were then re-tested for their effect on plant growth, chlorophyll content, nutrient uptake and eventually, on the metabolomic profile of plants.

### Partial characterization of the crude microalgae and cyanobacteria bio-extracts

Protein content in crude extracts was determined by using the 96 Well Plate Assay Protocol according to Bradford method^45^. The quantitative determination of total sugars was done according to the protocol described by^46^. The crude extracts were analysed subsequently for P-PO_4_, N-NO_3_, N-NH_4_ and K^+^ using a skalar nutrient auto analyser at MAScIR.

The biochemical composition of the general dry mass of microalgae and cyanobacteria was not studied, our focus mainly being projected on the effect/bio-stimulant properties of the end product of the biomass hydrolysis (Crude Extracts) of microalgae and cyanobacteria on plant morphological, physiological and biochemical mechanisms.

### Culture and treatment of tomato plants

The plant model used in this study was *Solanum lycopersicum* L. seeds, JANA F1 provided by BAYER Nunhems Netherlands BV. The Tomato seeds were sown on 24 cell seed trays filled with peat moss and were irrigated with distilled water. The seedling trays were then covered with aluminium foil to optimize germination and placed in a growth chamber at (MAScIR). The growth chamber conditions were set at 25 °C, 16:8 day/night photoperiod, 240 μmol photons m^−2^ s^−1^ and 60–70% relative humidity.

After germination (5 days after sowing), CBEs were applied as soil drench. Treatment with CBE was applied three times at an interval of 10 days. Also, two weeks after sowing, both treated and non-treated control plants were irrigated with 10 mL of Raukura nutrient solution every two days, alternated with distilled water. All plant cultures (Treated and non-treated control plants) were supplied with equal recommended doses of nutrients, according to^47,48^ Table 2. The resulting differences observed in N, P, K concentration and accumulation on the root biomass was therefore an indicator of their uptake by roots.

**Table 2:** The salts used to make up the nutrient solution.

Solution _A		Solution_B		Solution_C	
Element	g/L	Element	g/L	Element	mg/L
Mg (NO3)26H2O	4.94	KH2PO4	2.67	H3BO3	128.8
NH4NO	8.48	K2HPO4	1.64	CuCl2_2H2O	4.84
KNO3	2.28	K2SO4	6.62	MnCl2_4H2O	81.1
		Na2SO4	0.6	(NH4)6 Mo7O24_4H2O	0.83
		NaCl,	0.33	ZnCl2	23.45
				Ferric citrate pentahydrate	809.84

The applied dilute solution was prepared by mixing 100 mL of each of the macronutrient stock solution (A and B) with 50 mL of the micronutrient (solution C) supplement and diluting to 4.5 L with deionized distilled water. The pH was adjusted to 6.0 by adding HCl or KOH.

At 40 days old. The plants were harvested and measured for plant size. For each treatment, five replicates were dried in an oven at 50 °C while five other replicates were weighed and stoked at −80 °C.

### Peat moss Mixture

The floragard- “SPECIAL-MIXTURE” peat moss was obtained from Flora-gard Company, Floragard Vertriebs-GmbH.

**Origin:** Gebr.Brill Substrate GmbH & Co.KG – D-49828 Georgsdorf, Germany.

The physical-chemical characteristics of the peat moss are shown in Table 3.

**Table 3:** The Peat moss chemical and physical characteristics.

Major Constituents:	Mixture of weakly-moderate decomposed bog peat (H3-H5), strongly decomposed bog peat (H5-H8), with further additions like clay and perlite.	
Organic substances:	70–90% (Dry masses)	
Contents filling:	70L (EN 12580)	
Chemical properties:	pH (CaCl_2_):	5.7
	Salt g/L KCl	0.9
	N mg/L	140
	P_2_O_5_ mg/L	100
	K_2_O mg/L	180

### Measurement of the growth and pigments

The root and shoot lengths were measured manually with a ruler. Tomato leaves (0.1 g) sampled in the middle part of each plant with uniform size and color were homogenized in 4 mL of 80% acetone and 0.1% (w/v) of CaCO3 and left overnight at 4 °C. The pigment contents were measured using spectrophotometric technique and calculated using the method described by^49,50^

### Analysis of plant metabolomics profile using GC-MS

Extraction method was modified from the method previously described by^51^. 400 mg of the plant leaves was grinded in liquid nitrogen and transferred in a glass vial with a cap. 10 µl of internal standard Dodecane and 4 mL of CHCl_3_ (pre-cooled at −20 °C) were added to the vial. The mixture was thoroughly vortexed for 1 min. The vials were then transferred to a heat block (Labet International, Edison, USA) pre-set at 85 °C and left for 2hrs. After 2hrs, the vials were transferred to an ultrasound bath (Branson ultrasonic Sonifier 450, Danbury, USA) and sonicated at 60 °C for 1 hr. 2 mL of methanol was then added to the mixture. The vials were thoroughly vortexed, transferred back into the ultrasound bath and sonicated at 60 °C for 2 hrs. 1 mL of distilled water (dH_2_O) was then added to the vials (for phase separation), and the bottom organic phase was transferred to a fresh vial with the help of a separating funnel. The CHCl_3_ solvent was then evaporated completely under nitrogen flow. After that, 500 µl of 6% methanolic KOH (w/v) was added to the dried residue and the mixture was heated for 2 hrs at 85 °C and sonicated for 1 hr at 60 °C. The mixture was dried again under nitrogen flow, then, 250 µL of distilled H_2_O and 750 µL of CHCl_3_ was added to the dried residue and vortexed for 1 min. The bottom organic phase was transferred to a fresh vial with the help of a separating funnel and conserved at −20 for GC-MS analysis. **Note:** The mixtures were thoroughly vortexed for 1 min every 30 mins of the procedure.

Metabolomics analysis was carried out using gas chromatography (GC) (Agilent 7890A Series GC, USA) coupled to mass spectrometry (MS) equipped with multimode injector and BD-ASTMD6584 column (15 m × 0.320 mm × 0.1 µm) and electron impact ionization.

### Statistical analysis

Statistical analyses were performed by SPSS and R scripts. Descriptive statistics and statistically significant differences between the mean values from control and treated plant samples were determined using One-way ANNOVA and Turkey via SPSS (IBM SPSS statistics 22). Data presented are means ± standard errors of five replicates at the significant level of p < 0.05.

Statistical calculation and graphical generation were performed using the R programming environment. The PCA and heatmap was generated using RStudio, visualization of corrplot and ggplot packages, integrated into the R software. In order to perform the analysis, the data was centered and scaled. Principal component analysis PCA was calculated based on a Euclidean distance matrix and as a hierarchical ascendant classification (HAC) using the prcomp function and MixOmics package where each variable was centered by subtracting from the mean = True), but not by the standard deviation (scale = False). The first two components explained the maximum variance in the datasets (Table. S3).

## Supplementary information

Supplementary Information

## Data Availability

The data generated and/or analyzed during current study are available from the corresponding author on reasonable request
