# Supplementary material for: Screening of microalgae liquid extracts for their bio stimulant properties on plant growth, nutrient uptake and metabolite profile of Solanum lycopersicum L
Source: Sci Rep. 2020 Feb 18;10:2820. doi: 10.1038/s41598-020-59840-4 (PMC7028939; doi:10.1038/s41598-020-59840-4)
Supplement: Supplementary file 1 — Supplementary Information [file 41598_2020_59840_MOESM1_ESM.docx]

**Screening of microalgae liquid extracts for their bio stimulant properties on plant growth, nutrient uptake and metabolite profile of *Solanum lycopersicum* L.**

**Chanda Mutale-joan**1,3**, Benhima Redouane**1**, elmernissi Najib**1**, Kasmi Yassine**1**, Karim Lyamlouli**2**, Sbabou Laila**3**, Youssef Zeroual**2**, EL Arroussi Hicham**1**.**

S**1**: Selected microalgae and cyanobacteria crude extracts and their composition

| [Cyanobacteria](https://www.uniprot.org/taxonomy/1117) | **Extraction Solution** | **Applied Concentration** | **Sugar content (mgmL-1)** | **Protein content (mgmL-1)** | **N(mgL-1 )** | **N03-(mgL-1)** | **P(mgL-1)** | **K(mgL-1)** |
| --- | --- | --- | --- | --- | --- | --- | --- | --- |
| ***Aphanothese* sp.** | Sulfuric acid 0.1M | 0.5 gL-1 | 0.049 | 0.054 | 2.43 | 0.74 | 2.55 | 6.04 |
| ***Arthrospira maxima*** | Sulfuric acid 0.2M | 0.5 gL-1 | 0.048 | 0.078 | 3.94 | 0.87 | 5.11 | 46.35 |
| ***Arthrospira platensis*** | Distilled Water | 0.5 gL-1 | 0.038 | 0.168 | 3.92 | 1.9 | 4.77 | 36.27 |
| [Chlorophyta](https://www.uniprot.org/taxonomy/3041) |  |  |  |  |  |  |  |  |
| ***Chlorella pyrenoidosa*** | Sulfuric acid 0.1M | 0.1 gL-1 | 0.038 | 0.052 | 1.75 | 0.28 | 2.51 | 6.35 |
| ***Chlorella vulgaris*** | Sulfuric acid 0.2M | 0.5 gL-1 | 0.05 | 0.088 | 3.1 | 3.11 | 3.93 | 17.93 |
| ***Chlorella ellipsoidae*** | Sulfuric acid 0.1M | 1 gL-1 | 0.171 | 0.107 | 3.44 | 0.4 | 5.16 | 33.96 |
| ***Chlorella sorokiniana*** | Sulfuric acid 0.1M | 1 gL-1 | 0.083 | 0.13 | 3.32 | 1.32 | 2.98 | 15.23 |
| ***Chlorella marina*** | Sulfuric acid 0.1M | 0.5 gL-1 | 0.065 | 0.116 | 3.14 | 0.47 | 2.94 | 10.21 |
| ***Scenedesmus dimorphus*** | Sulfuric acid 0.2M | 0.5 gL-1 | 0.047 | 0.108 | 3.51 | 1.47 | 3.46 | 13.49 |
| ***Scenedesmus obliquus*** | Sulfuric acid 0.1M | 0.1 gL-1 | 0.014 | 0.017 | 1.58 | 0.25 | 3.6 | 7.9 |
| ***Chlamydomonas reinhardtii*** | Sulfuric acid 0.2M | 0.1 gL-1 | 0.025 | 0.026 | 1.55 | 0.03 | 2.46 | 12.34 |
| ***Dunaliella salina*** | Distilled Water | 0.5 gL-1 | 0.029 | 0.046 | 2.13 | 1.09 | 3.04 | 18.32 |
| ***Tetraselmis marina*** | Distilled Water | 0.5 gL-1 | 0.032 | 0.057 | 3.06 | 2.38 | 3.46 | 9.24 |
| ***Tetraselmis* sp.** | Sulfuric acid 0.2M | 0.5 gL-1 | 0.189 | 0.035 | 0.79 | 0.06 | 1.26 | 22.37 |
| ***Tetraselmis suecica*** | Distilled Water | 0.1 gL-1 | 0.029 | 0.052 | 3.06 | 0.62 | 1.36 | 11.69 |
| [Rhodophyta](https://www.uniprot.org/taxonomy/2763) |  |  |  |  |  |  |  |  |
| ***Porphyridium* sp.** | Distilled Water | 0.1 gL-1 | 0.025 | 0.008 | 0.32 | 0.05 | 0.82 | 6.7 |
| Haptophyta |  |  |  |  |  |  |  |  |
| ***Isochrysis galbana*** | Sulfuric acid 0.2M | 0.5 gL-1 | 0.019 | 0.024 | 3.31 | 0.04 | 2.52 | 24.65 |
| Ochrophyta |  |  |  |  |  |  |  |  |
| ***Nannochloropsis gaditana*** | Sulfuric acid 0.2M | 0.5 gL-1 | 0.239 | 0.162 | 2.24 | 1.15 | 2.58 | 29.69 |

S**2**: Generic function for plotting explained variance for data decomposition of plant growth parameters


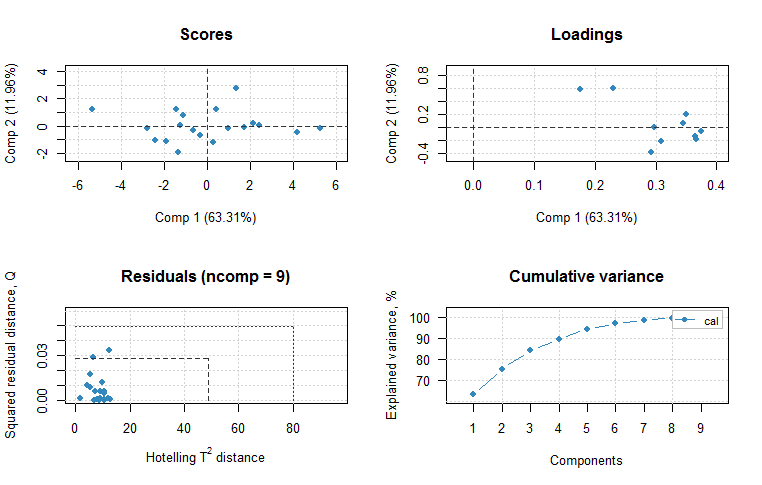


S**3**: Biplot (PCA) to understand treatment-variable relationships in treated tomato plants. The lines originating from central point of biplots indicate positive or negative correlations of different variables; where their closeness indicates correlation strength with particular treatment.


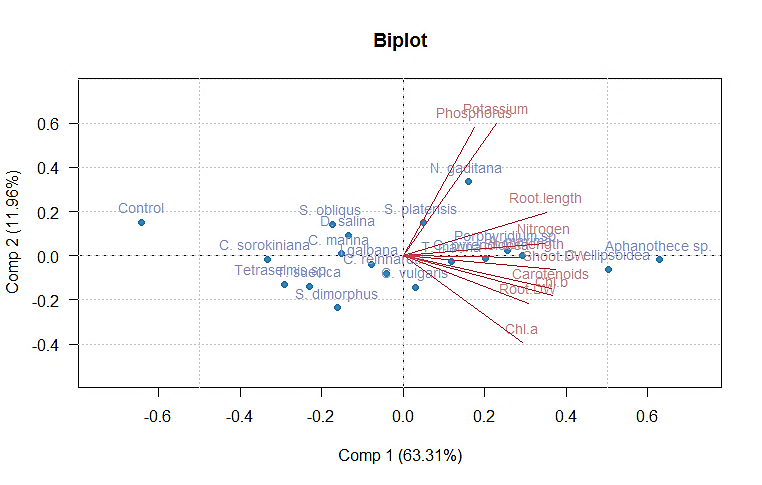


S**2**: Generic function for plotting explained variance for data decomposition of 17 metabolites detected in very high concentrations in some treated plants


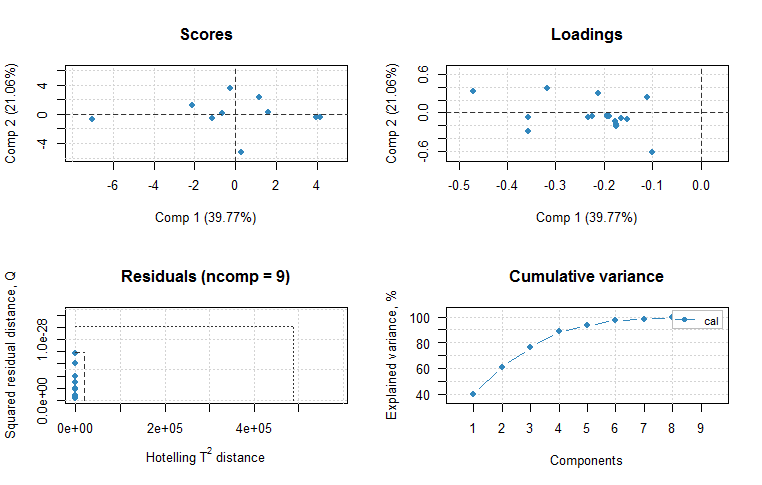


S**5**: Biplot (PCA) of normalized and centered data of 17 metabolites detected in very high concentrations in some treated plants. The lines originating from central point of biplots indicate positive or negative correlations of different variables; where their closeness indicates correlation strength with particular treatment. X1=Beta.-Sitosterol, X2=Linolenic acid, X3=Cetene, X4=Eicosane, X5=Arachidic acid, X6=Palmitic acid, X7=Naphthalene, X8=Neophytadiene, X9=Methyl palmitate, X10=Stearic acid, X11=Palmitoleic acid, X12=4-methoxyphenol, X13=Phytol, X14=Pyridine-3-carboxamide, X15=Myristic acid, X16=Triacontane, X17=Dihydroactinidiolide


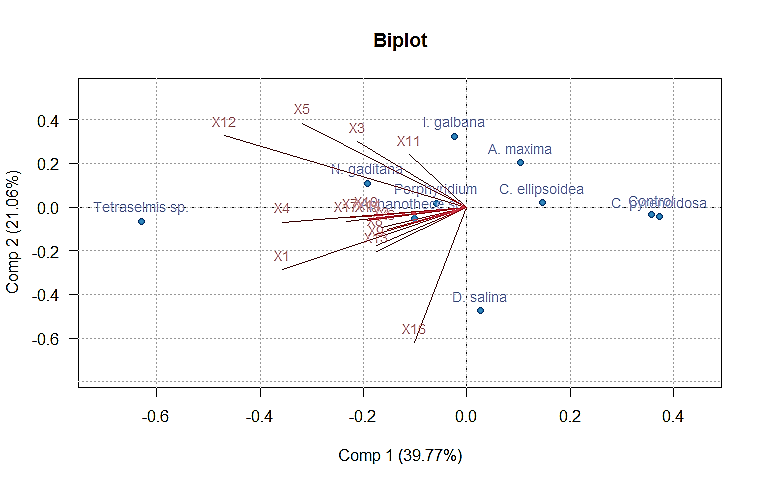


S**6**: Raw data of all detected metabolites in both treated and non-treated plants. Metabolites are expressed in µg/g.

|  | *Control* | *Aphanothece* sp. | *C. ellipsoidea* | *C. pyrenoidosa* | *A. maxima* | *Tetraselmis* sp. | *Porphyridium* sp. | *I. galbana* | *D. salina* | *N. gaditana* |
| --- | --- | --- | --- | --- | --- | --- | --- | --- | --- | --- |
| 3-Buten-2-one, 4-(2,6,6-trimethyl-1-cyclohexen-1-yl)- |  | 241.6588 |  |  |  |  |  |  |  |  |
| 4-tert-Butyl-2,6-diisopropylphenol |  |  |  |  |  |  |  |  |  | 1040.854 |
| Docosane |  |  |  |  |  | 7086.947 |  |  |  |  |
| (E)-3-Methyl-5-((1R,4aR,8aR)-5,5,8 a-trimethyl-2-methylenedecahydronaphthalen-1-yl)pent-2-en-1-ol |  |  | 72.62969 |  | 565.0484 |  |  |  |  |  |
| .alpha.-Amyrin |  |  |  |  |  |  |  |  | 4661.126 |  |
| .beta.-Amyrin | 285.4611 |  |  |  |  | 7672.219 |  |  |  |  |
| .beta.-Resorcylic acid, 3-(3,7-dimethyl-2,6-octadienyl)-6-pentyl-, ethyl ester, (E)- |  |  |  | 164.5215 |  |  |  |  | 6279.809 |  |
| .beta.-Sitosterol | 229.1063 | 7752.899 | 291.3078 |  |  | 39484.41 | 1448.425 | 317.6466 | 16568.47 | 7127.045 |
| 1,2-Benzisothiazole, 3-(hexahydro-1H-azepin-1-yl)-, 1,1-dioxide |  |  |  |  |  |  |  |  |  |  |
| 11,13-Dimethyl-12-tetradecen-1-ol |  |  |  | 561.9886 |  |  |  |  |  |  |
| 11,14,17-Eicosatrienoic acid, methyl ester |  |  |  | 125.493 |  |  |  |  |  |  |
| 11,14-Eicosadienoic acid, methyl ester |  |  |  |  |  |  |  |  | 1159.081 |  |
| 17-Pentatriacontene |  |  |  |  | 3541.991 |  |  |  | 2994.685 |  |
| 1-Bromo-11-iodoundecane |  |  |  |  |  |  | 596.8767 |  |  |  |
| 1-Chloroeicosane |  |  |  |  |  |  | 137.1869 |  |  |  |
| 1H-1,2,3-Triazolo[4,5-c]quinoline-1-hexanoic acid |  |  | 89.02394 |  |  |  |  |  |  |  |
| 1H-Inden-1-one, 2,3-dihydro-5,6-dimethoxy-3-methyl- |  | 3177.999 | 229.356 | 185.4663 | 1572.947 | 36794.07 | 1777.559 | 1734.322 | 36590.85 | 7481.302 |
| 1-Hydroxymethyl-2-methyl-1-cyclohe |  |  |  |  |  |  |  |  | 21793.28 |  |
| 1-Nonadecene | 25.28861 | 3056.94 |  |  |  |  |  | 981.3031 |  | 4539.11 |
| 1-Octadecene | 36.78564 | 302.5859 |  | 296.6074 |  | 4574.247 |  |  | 1281.027 | 745.1302 |
| 1-Pentadecanethiol |  | 2858.678 |  |  |  |  |  |  |  |  |
| 1-Pentadecene |  |  |  |  |  |  | 60.18287 |  |  |  |
| 1-Tetradecene |  | 118.6464 |  |  |  |  |  |  |  |  |
| 2(4H)-Benzofuranone, 5,6,7,7a-tetrahydro-4,4,7a-trimethyl-, (R)- |  | 551.0312 | 51.95438 | 67.37568 | 404.0198 | 3271.098 | 152.6628 | 212.4123 | 1372.94 | 698.2032 |
| 2,3-Dihydroxybenzoic acid, 3TMS de rivative |  |  |  | 63.82096 |  |  |  |  |  |  |
| 2,4-Di-tert-butylphenol | 232.5835 | 4161.994 |  | 552.8939 | 3261.154 | 56647.47 |  | 179.7697 |  | 9078.247 |
| 2,5,5,8a-Tetramethyl-4-methylene-4 a,5,6,7,8,8a-hexahydro-4H-chromene |  |  |  |  |  |  | 317.6466 |  |  |  |
| 2,6-Nonadienoic acid, 7-ethyl-9-(-ethyl-3-methyloxiranyl)-3-methyl-, methyl ester |  |  |  |  |  |  |  |  | 5097.335 |  |
| 2H-Pyran-2-one, tetrahydro-6-undecyl- |  |  |  |  |  | 10464.03 |  |  |  |  |
| 2-Methyltriacontane |  | 2938.158 |  |  | 3117.119 |  |  |  |  | 4832.764 |
| 2-Tetradecene, (E)- |  |  |  | 29.64862 |  |  |  |  |  |  |
| 3,5-di-tert-Butyl-4-hydroxybenzald ehyde |  |  |  |  |  |  | 307.8147 | 186.9367 |  |  |
| 3,5-Di-tert-butylbenzoic acid | 53.75233 |  |  | 16.05036 |  | 6902.23 | 186.9367 |  |  |  |
| 3-Eicosene, (E)- |  |  |  |  |  |  | 210.8205 |  |  |  |
| 3-Heptadecen-5-yne, (Z)- |  | 2258.286 |  |  |  |  |  |  |  |  |
| 4-tert-Butyl-2,6-diisopropylphenol |  |  |  |  | 428.357 |  |  | 45.25622 | 1300.14 |  |
| 5-Octadecene, (E)- | 76.09791 |  | 33.9134 |  |  |  |  |  |  |  |
| 6-C14H26 |  |  |  |  | 3017.262 |  |  |  |  |  |
| 6-Methoxy-3-methyl-2-benzofurancarboxylic acid |  |  |  |  |  |  | 616.984 |  |  |  |
| 7,10,13-Hexadecatrienoic acid, methyl ester |  |  |  |  | 1701.088 | 19302.58 |  | 91.83755 | 4813.199 | 3004.638 |
| 7,9-Di-tert-butyl-1-oxaspiro(4,5)deca-6,9-diene-2,8-dione |  |  | 217.5322 |  |  |  | 598.7851 |  |  |  |
| 9,10-Secocholesta-5,7,10(19)-triene-3,25,26-triol, (3.beta.,5Z,7E)- |  |  |  |  |  |  |  |  |  | 2017.497 |
| 9,12,15-Octadecatrienoic acid, (Z,Z,Z)- | 29791.2 | 230462 | 20620.49 | 18623.79 | 197061.9 | 1574664 | 72433.69 | 69324.91 | 681316.6 | 261634.1 |
| 9,19-Cyclolanost-25-en-3-ol, 24-methyl-, (3.beta.,24S)- |  |  |  | 171.9976 |  |  |  |  |  |  |
| 9-Eicosene, (E)- |  |  | 67.378 | 36.87772 |  |  |  |  |  |  |
| 9-Hexadecenoic acid |  |  |  |  |  |  |  |  |  | 4973.399 |
| 9-Octadecene, (E)- |  |  | 18.40764 |  |  |  |  |  |  |  |
| Behenyl chloride |  | 402.6387 |  |  |  |  |  |  |  |  |
| Carbonic acid, decyl undecyl ester |  |  |  |  |  |  |  |  |  | 301.8765 |
| Cetene |  |  |  | 97.36211 | 472.0393 | 2470.643 | 142.0787 | 279.4461 |  | 217.0516 |
| Chavicol TMS |  |  |  |  |  |  |  |  | 2508.116 |  |
| Cholesterol |  | 848.709 |  |  |  |  |  |  |  |  |
| cis-13-Octadecenoic acid |  |  |  |  |  |  | 321.2382 |  |  |  |
| Cyclodecane |  |  |  |  | 409.2577 |  |  |  |  |  |
| Cyclododecyne |  | 1319.783 |  |  |  | 14556.68 |  |  |  | 2043.83 |
| Cyclohexadecane, 1,2-diethyl- | 39.72352 | 383.0407 |  |  |  |  |  |  |  |  |
| Cyclohexanol, 5-methyl-2-(1-methylethenyl)- |  |  | 84.04872 |  |  |  |  |  |  |  |
| Cyclooctene, 3-ethenyl- |  |  | 333.2788 |  |  |  | 431.0109 | 5375.969 |  |  |
| Cyclotetracosane |  |  |  |  | 3392.987 |  |  |  |  |  |
| Cyclotriacontane |  |  | 292.3364 |  |  |  |  |  |  |  |
| Dantrolene |  |  |  |  | 2094.3 |  |  |  |  |  |
| Decane, 1-iodo- |  |  | 26.38054 |  |  |  |  |  |  |  |
| Disulfide, di-tert-dodecyl |  | 832.8348 | 63.99614 |  |  |  | 91.83755 |  |  |  |
| dl-.alpha.-Tocopherol |  |  | 23.60322 |  |  |  |  |  |  |  |
| Docosane |  | 571.9576 | 15.01857 |  | 229.9745 |  |  |  |  |  |
| Dodecanoic acid |  | 366.4122 | 54.33593 |  | 293.7225 | 2146.379 | 134.9735 | 152.6628 |  | 1002.326 |
| Dotriacontane | 369.0052 |  |  |  |  | 23344.77 | 1179.693 |  |  | 8355.074 |
| E-15-Heptadecenal |  |  |  |  | 479.2381 |  |  |  |  |  |
| Eicosane | 1420.201 | 3893.806 | 1142.268 | 97.40757 | 2308.78 | 82620.32 | 117.279 | 512.7965 | 1788.499 | 16499.04 |
| Eicosane, 2,6,10,14,18-pentamethyl |  |  |  |  |  | 48954.36 |  |  |  |  |
| Eicosanoic acid | 148.7047 | 2142.406 | 358.385 |  | 3387.42 | 34816.73 | 960.0619 | 1477.178 |  | 4481.962 |
| Ethanol, 2-(tetradecyloxy)- |  |  |  |  | 2120.81 |  |  |  |  |  |
| Ethyl 9,12,15-octadecatrienoate |  |  |  |  |  |  |  |  | 4415.878 |  |
| Heneicosane |  | 23.13358 | 277.4558 | 362.1923 | 2191.125 | 16462.41 |  | 745.9279 |  | 2108.186 |
| Heneicosane, 11-cyclopentyl- |  |  |  | 255.5619 |  |  |  |  | 9946.768 |  |
| Heneicosane, 11-decyl- |  |  |  |  |  | 1864.628 |  |  |  |  |
| Hentriacontane |  | 18770.95 |  | 1219.382 | 16849.02 | 6669.636 | 3722.109 | 3327.88 | 38311.88 |  |
| Heptacosane |  |  | 215.2771 |  | 1866.231 |  |  | 4452.348 |  |  |
| Heptacosane, 1-chloro- | 58.51567 |  | 31.38798 |  |  |  |  |  |  | 7324.425 |
| Heptadecane |  |  | 61.18482 | 113.6983 | 11079.46 | 51404.2 | 477.3479 | 321.2382 |  |  |
| Heptadecane, 2-methyl- |  |  |  |  |  |  |  |  |  | 438.4211 |
| Heptadecanolide |  |  |  |  |  |  |  | 431.0109 |  |  |
| Hexacosane |  | 272.1383 | 166.6432 |  |  | 2385.582 |  |  |  | 3022.189 |
| Hexadecane |  |  | 181.2726 |  |  |  |  |  |  |  |
| Hexadecane, 1-iodo- | 249.8337 |  | 691.2734 | 727.5992 | 2150.743 | 24554.29 |  | 267.9246 |  |  |
| Hexadecane, 2,6,10,14-tetramethyl- |  |  |  |  | 8833.943 |  |  |  |  |  |
| Hexadecanoic acid, methyl ester | 118.0551 |  | 179.7778 | 131.1458 | 1690.757 | 17978.87 | 686.0769 | 132.9978 | 6449.661 | 2927.591 |
| Hop-22(29)-en-3.beta.-ol |  |  |  |  |  |  |  | 84.18049 | 5159.398 |  |
| i-Propyl 9,12,15-octadecatrienoate |  |  |  |  |  | 15508.37 |  |  |  |  |
| Linoelaidic acid | 30.88875 |  |  | 296.0158 |  |  |  |  |  |  |
| Lupeol |  |  |  |  |  | 10266.99 | 443.5508 |  |  | 2019.262 |
| Methyl 8,11,14-heptadecatrienoate |  |  | 377.0415 | 105.0929 | 5708.045 | 22222.58 |  |  |  |  |
| Methyl stearate |  |  |  |  |  |  |  |  |  | 2808.164 |
| Molybdenum, dicarbonyl-(.eta.-2-cyclooctene)-(hexamethylbenzene) |  |  |  |  |  |  |  |  | 5115.482 |  |
| Naphthalene, 1,2,3,4-tetrahydro-1,1,6-trimethyl- |  | 463.8131 | 22.57232 | 85.9926 | 436.3568 | 2527.49 | 179.7697 | 184.9085 | 911.6228 | 647.0776 |
| Neophytadiene | 1729.997 | 14022.46 | 841.0558 | 1146.77 | 11771.1 | 73165.55 | 2767.199 | 891.1661 | 47971.5 | 125453.8 |
| n-Hexadecanoic acid methyl ester | 6818.108 | 83929.86 | 7831.436 | 6761.925 | 76161.13 | 639073 | 28205.76 | 2133.238 | 286094 | 110769.3 |
| Nonacosane |  |  |  |  | 710.2066 | 3399.597 |  |  |  |  |
| Nonadecane |  |  |  |  | 2335.446 |  | 427.0597 |  |  |  |
| Nonadecane, 1-chloro- |  |  | 20.64765 |  |  |  |  |  |  |  |
| Nonadecane, 2,6,10,14-tetramethyl- |  |  |  |  |  |  | 308.2584 |  |  |  |
| Nonadecane, 9-methyl- |  |  |  |  |  |  |  |  | 1430.628 |  |
| n-Tetradecyltrichlorosilane |  |  |  |  |  |  |  |  |  | 1562.79 |
| Octacosane | 227.5455 | 1945.432 | 446.854 | 1005.425 |  | 17290.59 | 17290.59 | 1995.414 |  |  |
| Octadecanal |  |  |  |  |  | 13025.15 |  |  |  | 1854.55 |
| Octadecanal |  |  |  |  |  |  |  |  |  |  |
| Octadecane |  |  |  |  |  |  |  |  |  | 9319.321 |
| Octadecane, 1-(ethenyloxy)- |  |  |  |  |  | 19072.06 |  |  |  |  |
| Octadecane, 1-chloro- |  |  | 296.0361 |  |  |  |  |  |  |  |
| Octadecane, 1-iodo- |  |  |  |  | 4493.648 | 51797.42 |  | 518.5346 | 34321.66 | 627.3415 |
| Octadecane, 2,6,10,14-tetramethyl- |  |  |  |  |  |  |  | 514.422 |  |  |
| Octadecanoic acid | 3418.002 | 28527.18 | 5165.833 | 2535.268 | 42302.67 | 431029.8 | 12852.62 | 28205.76 | 93498.8 | 83507.13 |
| Oleyl alcohol, trifluoroacetate |  | 717.1053 |  |  |  |  |  |  | 3491.327 |  |
| Oxirane, tridecyl- |  |  |  |  | 1295.408 |  |  |  |  |  |
| Palmitoleic acid | 230.2747 | 3571.072 | 389.34 | 328.5665 | 3438.46 | 32055.59 | 1303.627 | 633.9611 |  |  |
| Pentacosane |  | 1249.187 | 104.7153 |  |  | 23919.61 | 664.5443 |  | 4470.843 |  |
| Pentadecafluorooctanoic acid, hexadecyl ester |  |  |  | 179.8542 |  |  |  |  |  |  |
| Pentafluoropropionic acid, dodecyl |  | 499.7246 |  |  |  |  |  |  |  |  |
| Phenanthrene, 9,10-dimethyl |  |  |  |  |  |  |  |  |  | 2268.266 |
| Phenanthrene, 9,10-dimethyl- |  |  |  |  |  |  |  | 137.1869 |  |  |
| Phenol, 2-(1,1-dimethylethyl)-4-(,1,3,3-tetramethylbutyl)- |  |  |  |  |  | 16713.89 |  |  |  |  |
| Phenol, 2,5-bis(1,1-dimethylethyl) |  |  |  |  |  |  | 2550.201 |  | 14514.83 |  |
| Phenol, 2,6-bis(1,1-dimethylethyl)-4-ethyl- |  | 449.3567 | 959.2226 |  |  |  |  |  |  |  |
| phenol, 4-methoxy-2-[2-(5-nitro-2- |  |  |  |  |  |  |  |  | 15902.26 |  |
| phenol, 4-methoxy-2-[2-(5-nitro-2-pyridinyl)diazenyl]- |  | 3352.183 | 446.9196 |  |  | 41222.94 | 1477.178 | 22729.05 |  | 6470.425 |
| Phytol | 2420.654 | 51904.88 | 5177.52 | 7784.873 | 50181.43 | 418997.1 | 21592.42 | 686.0769 | 231422.7 | 82567.87 |
| Phytyl palmitate | 695.3591 | 7652.002 |  |  | 8197.526 |  |  |  |  |  |
| Pregnane-3,11,20,21-tetrol |  |  |  | 140.3621 |  |  |  |  |  |  |
| Pyridine-3-carboxamide, oxime, N-(2-trifluoromethylphenyl)- | 125.5562 | 1946.175 | 293.4621 | 0.204685 | 3480.941 | 13696.35 | 1817.228 | 3907.74 | 19244.22 | 6956.767 |
| Santolina epoxide | 307.077 |  |  |  |  |  |  |  |  |  |
| Stigmasta-5,24(28)-dien-3-ol, (3.beta.,24Z)- |  |  | 129.3795 |  | 1299.692 |  |  |  |  |  |
| Tetracosane | 42.47921 | 5933.491 | 601.0538 |  | 450.2018 | 1732.998 |  | 960.0619 |  |  |
| Tetradecanal |  | 1347.176 |  |  |  |  | 466.0724 |  |  |  |
| Tetradecane |  |  |  |  |  |  | 45.25622 |  |  |  |
| Tetradecane, 2,6,10-trimethyl- |  | 343.1146 |  |  |  |  |  |  |  |  |
| Tetradecanoic acid | 28.06232 | 457.8593 | 59.34522 | 30.36456 | 470.1948 | 4313.425 | 132.9978 | 210.8205 | 1166.895 | 701.0879 |
| Thunbergol |  |  | 337.7064 |  |  |  |  |  |  |  |
| Triacontane | 403.4937 | 5481.694 | 764.4335 | 1226.816 |  | 35866.45 | 2998.749 |  | 29484.04 |  |
| Tricosane | 144.76 |  | 443.4328 |  |  | 23665.47 | 290.9154 | 522.5643 | 4508.246 | 12426.04 |
